# Visibility-Guided and Occlusion-Simulated Learning for Robust Person Re-Identification

**DOI:** 10.3390/s26103137

**Published:** 2026-05-15

**Authors:** Junjie Cao, Rong Rong, Xing Xie

**Affiliations:** 1 School of Artificial Intelligence and Computer Science, Nantong University, Nantong 226019, China; 2330110408@stmail.ntu.edu.cn (J.C.); rongrong@stmail.ntu.edu.cn (R.R.); 2 Engineering Training Center, Nantong University, Nantong 226019, China

**Keywords:** occluded person re-identification, visible-region modeling, fine-grained feature learning, occlusion-aware learning

## Abstract

Occlusion is a critical challenge in person re-identification (ReID), as partial visibility severely degrades feature discriminability and matching reliability. To address this issue, we propose a novel framework termed Visibility-Guided and Occlusion-Simulated Learning (VGOSL) for robust person ReID. The framework consists of two key components: a part-aware visibility modeling (PVM) module and an occlusion box simulation (OBS) module. The PVM module explicitly estimates part-level visibility reliability and adaptively reweights local features to guide global representation learning, enabling the model to emphasize informative regions while suppressing occluded ones. Meanwhile, the OBS module introduces structured occlusion box simulation during training to enhance robustness against realistic obstruction patterns through multi-branch supervision. Extensive experiments on Occluded-DukeMTMC, DukeMTMC-reID, Market-1501, Partial-ReID, and MSMT17 demonstrate that the proposed framework achieves competitive performance under both occluded and holistic settings. The source code has been publicly released on GitHub.

## 1. Introduction

Person re-identification aims to associate pedestrian images captured across non-overlapping camera views and serves as a fundamental component of intelligent surveillance systems. With the emergence of large-scale datasets such as Market-1501 and MSMT17, deep learning-based approaches have significantly advanced the field. Early CNN-based models, including IDE [1], PCB [2], and MGN [3], improve representation learning through part-based modeling and multi-granularity supervision. More recently, Transformer-based architectures, such as TransReID [4], PVT-based ReID [5], and ViT-based adaptations [6], further enhance global context modeling and achieve state-of-the-art performance under holistic settings. Despite these advances, occlusion remains a critical yet underexplored challenge in practical scenarios. In crowded environments, pedestrians are frequently partially occluded by other individuals, vehicles, or scene objects, leading to incomplete visual observations and corrupted feature representations. Performance degradation under occlusion has been widely observed on dedicated benchmarks such as Occluded-DukeMTMC and Partial-ReID.

**Figure 1:**
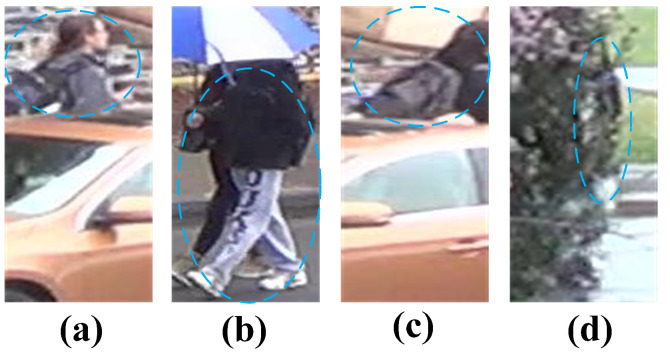
Illustration of challenging occluded person images. The blue circles indicate the regions of interest emphasized in our comparative analysis. In (**a**,**b**), discriminative cues mainly lie in local visible regions, while in (**c**,**d**), severe occlusion introduces strong interference and noise.

As shown in Figure 1, occluded person re-identification is challenged by both local discriminative dependency and severe occlusion interference. In cases (a) and (b), the key identity cues mainly lie in local regions, and relying too much on global information may mislead the model. In cases (c) and (d), the pedestrians are heavily occluded, so without proper handling, the model can be easily affected by noise and irrelevant regions. These examples illustrate the importance of both local feature modeling and occlusion-robust learning.

To address partial visibility, several studies propose part-alignment or region-selection strategies. Partial ReID [7] introduces region matching for incomplete body images, while APH [8] leverages alignment and hallucination mechanisms. Visibility-aware models such as VPM [9] explicitly detect visible regions for matching, and SCPNet [10] introduces spatial-channel parallel learning to enhance occlusion robustness. Other works, including PGFA [11], HOReID [12], and PAT [13], attempt to model part-level correlations or graph relationships for robust representation learning. Additionally, data augmentation strategies such as random erasing [14], Cutout [15], and DropBlock [16] are widely adopted to simulate occlusion during training.

Although these methods improve robustness to some extent, two fundamental limitations remain. First, most approaches rely on implicit attention mechanisms to handle occlusion without explicitly supervising part-level visibility reliability. As a result, occluded regions may still introduce noisy or misleading information during feature aggregation. Second, existing occlusion simulation strategies are typically random and unstructured, lacking controllability and semantic consistency. Random erasing-based methods cannot accurately reflect realistic obstruction patterns, thereby limiting generalization to real-world scenarios.

To overcome these challenges, we propose a unified framework termed Visibility-Guided and Occlusion-Simulated Learning (VGOSL) for robust person re-identification. The framework integrates two complementary components.

Our design is motivated by the observation that occluded person ReID requires not only local part modeling but also reliable estimation of which parts remain informative under occlusion. Existing part-based methods improve discriminative representation through local region partition and part-level feature learning, while adaptive feature weighting strategies enhance informative responses during feature aggregation. However, these mechanisms usually do not explicitly model the visibility reliability of each local part under occlusion. Based on this observation, our framework explicitly estimates part-level visibility reliability and further combines it with structured occlusion simulation to guide robust feature learning.

First, we design a part-aware visibility modeling (PVM) module to explicitly estimate the reliability of local part representations. By introducing patch-level visibility supervision, the model learns to predict part visibility scores and adaptively reweight local features during global aggregation. This mechanism suppresses unreliable regions while emphasizing informative parts, leading to more stable and discriminative representations under occlusion.

Second, we introduce an occlusion box simulation (OBS) module that generates structured and controllable occlusion patterns during training. Unlike random erasing-based strategies, OBS simulates realistic obstruction via structured occlusion boxes and adopts multi-branch supervision to encourage occlusion-invariant feature learning. The joint optimization of PVM and OBS enables the model to learn visibility-aware and occlusion-robust representations in a unified manner.

Extensive experiments on five widely used benchmarks, including Occluded-DukeMTMC, DukeMTMC-reID, Market-1501, Partial-ReID, and MSMT17, demonstrate that the proposed framework achieves competitive performance under both holistic and occluded settings, validating the effectiveness and generalization capability of explicit visibility modeling and structured occlusion simulation. The main contributions of this work are summarized as follows:

- We propose a unified occlusion-robust ReID framework, VGOSL, integrating visibility-aware feature aggregation and structured occlusion simulation.
- We design a part-aware visibility modeling (PVM) module that explicitly supervises part-level reliability and guides adaptive global representation learning.
- We introduce an occlusion box simulation (OBS) module to generate structured and controllable occlusion patterns for robust multi-branch learning.
- Extensive evaluations on both holistic and occlusion benchmarks verify the effectiveness and generalization capability of the proposed approach.

The remainder of this paper is organized as follows: Section 2 RELATED WORK reviews related work on occlusion-robust person re-identification and occlusion simulation strategies. Section 3 Methods presents the proposed VGOSL framework, including the PVM and OBS modules as well as the overall training objective. Section 4 EXPERIMENTS reports the experimental settings, comparison results, ablation studies, and visualization analyses. Finally, Section 5 concludes this paper.

## 2. Related Work

### 2.1. Occlusion-Robust Person Re-Identification

In recent years, research on occlusion-robust person re-identification has gradually shifted from alignment and selection toward visibility reasoning and relational modeling. One line of work attempts to recover occluded information through feature completion or dynamic denoising, such as learning frameworks based on feature diffusion and erasing [17], as well as Transformer-based models for feature completion [18]. Another line emphasizes inter-part relations and prototype-mask modeling, using graph attention or dynamic prototype mechanisms to select and enhance visible patterns [19,20]. In addition, methods that incorporate human parsing or keypoint guidance for alignment and ambiguity reduction have also significantly improved robustness in occluded scenarios [21,22]. Recent studies have also extended person ReID to more challenging unsupervised lifelong settings. For example, AHN [23] has been introduced to alleviate distribution inconsistency across training stages and improve continual adaptation capability. Although these approaches have achieved notable progress under occlusion, most of them still rely on implicit attention or external alignment cues to alleviate occlusion interference, while the reliability of local region visibility often lacks explicit supervision. Visibility estimation is usually decoupled from global feature aggregation, which allows occluded regions to still introduce noise during the fusion process.

To address this issue, we propose a part-aware visibility modeling (PVM) module, which explicitly predicts part-level visibility scores within an end-to-end framework and directly uses them for adaptive reweighting of global features. Compared with existing methods, PVM unifies visibility estimation, feature reweighting, and global aggregation into a single modeling process, thereby suppressing the interference of unreliable regions on identity discrimination at the mechanism level.

### 2.2. Occlusion Simulation and Data Augmentation Strategies

Beyond architectural design, occlusion simulation and data augmentation have also become a key pathway for improving robustness. Traditional methods, such as random erasing and its variants, can alleviate overfitting to some extent, but the generated occlusion patterns are usually random and lack structural constraints. In recent years, researchers have begun to introduce more semantically consistent occlusion modeling strategies, such as training mechanisms based on dynamic mask selection [20], occlusion augmentation frameworks combined with feature diffusion [17], and multi-branch interactive learning schemes to strengthen information fusion under occluded conditions [24].

In addition, some studies have extended the problem to more complex scenarios, such as joint task settings that simultaneously consider occlusion and clothing changes [25], or multi-granularity consistency learning methods that enhance cross-modality matching under occlusion [26]. In heterogeneous ReID settings, recent work has further explored unsupervised visible-infrared person re-identification, where BIMM [27] is adopted to enhance semantic alignment and inter-modality consistency without manual annotations. More recent works have further explored second-order attention and hierarchical structure modeling to improve discriminative capability under complex occlusion relationships [28].

However, most existing augmentation strategies still generate occluded regions in a random or weakly structured manner, lacking controllability and consistency with human body layout, which makes it difficult to realistically simulate occlusion patterns encountered in real-world scenarios. To address this limitation, we propose an occlusion box simulation (OBS) module, which simulates realistic occlusions through a structured and controllable occlusion-box generation mechanism, and is jointly optimized with multi-branch supervision.

## 3. Methods

### 3.1. Overview

**Figure 2:**
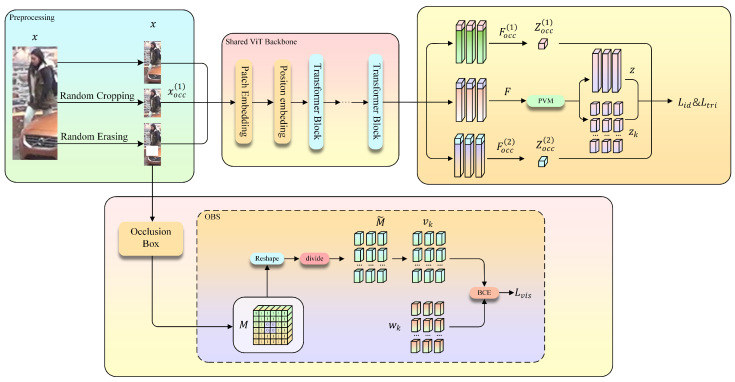
Overall framework of the proposed VGOSL for occlusion-robust person re-identification. The original and occluded images are first fed into a shared Transformer backbone to extract global and part-aware features. Based on the extracted part features, the proposed PVM estimates part reliability and performs visibility-guided feature aggregation to obtain the occlusion-robust person representation. Meanwhile, the OBS module generates two occluded counterparts during preprocessing and provides occlusion-related supervision for training.

We propose VGOSL, a unified framework for occlusion-robust person re-identification. The overall pipeline of the proposed method is shown in Figure 2. Given a training image *x*, we generate two occluded counterparts $\left\{x_{occ}^{\left(1\right)},x_{occ}^{\left(2\right)}\right\}$ during the preprocessing stage. Specifically, $x_{occ}^{\left(1\right)}$ is obtained via random resized cropping, which simulates partial visibility by spatial truncation. $x_{occ}^{\left(2\right)}$ is generated using random erasing with a sampled occlusion box, where a rectangular region is replaced with random noise. These three images are forwarded through a shared Transformer backbone to obtain global and part-aware representations. To mitigate the influence of occluded regions, we introduce PVM, which explicitly estimates part-level visibility reliability and uses it to guide global feature aggregation. Meanwhile, the erasing operation provides the corresponding occlusion box coordinates. Based on these coordinates, the OBS module constructs a binary patch-level visibility mask $M\in {\left\{0,\;1\right\}}^{H\times W}$ indicating visible and occluded regions. The mask *M* is used for explicit supervision of part reliability during training.

### 3.2. Baseline

We adopt a Transformer-based ReID architecture as our baseline, instantiated by a ViT-B/16 [6] backbone with SIE embeddings for camera/view labels [4]. Let f(·) denote the shared backbone. For an input image *x*, the backbone outputs a sequence of tokens:

(1) $$F=f\left(x\right),F_{occ}^{\left(1\right)}=f\left(x_{occ}^{\left(1\right)}\right),F_{occ}^{\left(2\right)}=f\left(x_{occ}^{\left(2\right)}\right)\in R^{(1+N)\times C},$$

where the first token is the [cls] token, the remaining *N* tokens correspond to patch embeddings, and C denotes the channel dimension of each token feature. The global representations are directly obtained by taking the [cls] token from the backbone outputs of *x*, $x_{occ}^{\left(1\right)}$, and $x_{occ}^{\left(2\right)}$, producing $Z_{g}$, $Z_{occ}^{\left(1\right)}$ and $Z_{occ}^{\left(2\right)}$, respectively.

### 3.3. PVM: Part-Aware Visibility Modeling

**Figure 3:**
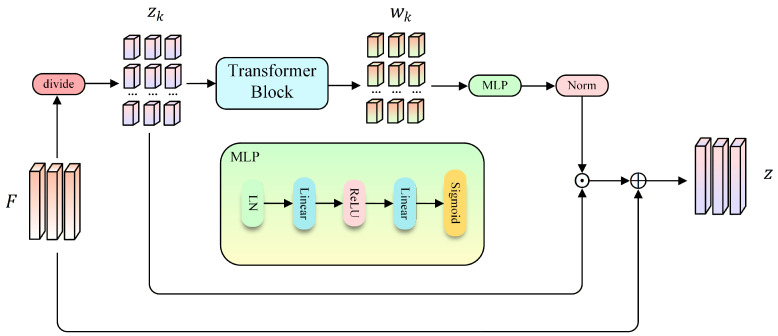
Overview of the proposed PVM module. The module predicts part reliability scores for local features, performs weighted part aggregation, and combines the resulting part-aware representation with the global feature to produce the final representation.

To enhance robustness against partial occlusion, we introduce a PVM module that further decomposes the patch tokens in *F* into multiple local groups, as illustrated in Figure 3. Since occlusion typically affects only a subset of body regions, learning representations at a finer spatial granularity allows the model to preserve discriminative cues from visible parts while mitigating the impact of corrupted regions. Specifically, based on the patch tokens in *F*, we divide the *N* tokens into *K* non-overlapping local groups. Let $\Omega_{k}$ denote the token index set of the *k*-th group, and $P_{k}=\left\{t_{i}|i\in \Omega_{k}\right\}$ denote the corresponding local token subset. Optionally, a token rearrangement operator is applied to increase local diversity and reduce positional bias. For the *k*-th group $P_{k}$, we prepend the class token $t_{cls}$ and feed the resulting sequence into the final Transformer block to obtain a part-level representation:

(2) $$z_{k}=h{\left(\left[t_{cls};P_{k}\right]\right)}_{cls}\in R^{C},\;k=1,\ldots ,K,$$

where h(·) denotes the final Transformer block. However, since occlusion mainly affects local regions, treating all parts equally during aggregation may introduce noisy cues. To address this, we estimate the visibility of each part feature, and use it to guide the global representation. Specifically, given part features ${\left\{z_{k}\right\}}_{k=1}^{K}$, PVM predicts a scalar visibility score $w_{k}\in \left(0,\;1\right)$ for each part through a lightweight gating network:

(3) $$w_{k}=\sigma \left(\phi \left(z_{k}\right)\right),$$

where ϕ(·) is implemented as a two-layer MLP with Layer Normalization and ReLU activation, and σ(·) is the sigmoid function. We then normalize the scores across parts:

(4) $${\hat{w}}_{k}=\frac{w_{k}}{\sum_{j=1}^{K}w_{j}+\epsilon },$$

where ϵ is a small positive constant set to $1\times 10^{-6}$ for numerical stability. Then, we compute a part-aware global feature by weighted aggregation:

(5) $$z_{p}=\sum_{k=1}^{K}{\hat{w}}_{k}\;z_{k},$$

Finally, we fuse it with the baseline global feature by a learnable scaling parameter α:

(6) $$z=Z_{g}+\alpha z_{p},$$

### 3.4. OBS: Occlusion Box Simulation Module

To enhance robustness against partial occlusion, we introduce an occlusion box simulation (OBS) module that explicitly generates structured occluded samples during training, as illustrated in Figure 2. For each input image, a rectangular occlusion box is randomly sampled with stochastic area and aspect ratios, and the selected region is replaced with random noise to produce an occluded counterpart.

More importantly, the sampled occlusion box provides explicit supervision for part visibility learning. Instead of using only image-level augmentation, OBS further converts the simulated occlusion region into a patch-level visibility mask. Specifically, let $M^{p}\in {\left\{0,\;1\right\}}^{H_{p}\times W_{p}}$ denote the binary visibility mask on the transformer patch grid, where 0 indicates an occluded patch and 1 indicates a visible patch. Here, $H_{p}$ and $W_{p}$ denote the height and width of the patch grid, respectively.

To align the mask with the token sequence of the Transformer backbone, we flatten $M^{p}$ in row-major order as

(7) $$\tilde{M}=\mathrm{vec}\left(M^{p}\right)\in {\left\{0,\;1\right\}}^{N},\;N=H_{p}W_{p},$$

where vec(·) denotes the row-wise flattening operation that reshapes the 2D patch-level mask into a 1D vector, and $\tilde{M}$ follows the same ordering as the patch tokens in the backbone output. Then, according to the same token partition strategy used in the part branch, the flattened mask is divided into *K* contiguous part groups. Let $\Omega_{k}$ denote the token index set of the *k*-th part group. The ground-truth visibility ratio of each part is computed by averaging the mask values within the corresponding token group:

(8) $$v_{k}=\frac{1}{|\Omega_{k}|}\sum_{i\in \Omega_{k}}{\tilde{M}}_{i},\;k=1,\ldots ,K,$$

where $v_{k}\in \left[0,\;1\right]$ represents the visible proportion of the *k*-th local part. In this way, the simulated occlusion pattern is converted into explicit part-level soft supervision.

Given the predicted part reliability logits ${\left\{w_{k}\right\}}_{k=1}^{K}$ from PVM, we impose a visibility supervision loss to encourage consistency between the predicted reliabilities and the actual visible proportions:

(9) $$L_{\mathrm{vis}}=-\frac{1}{K}\sum_{k=1}^{K}\left[v_{k}\log\left(w_{k}\right)+\left(1-v_{k}\right)\log\left(1-w_{k}\right)\right].$$

Through OBS, the model not only encounters more diverse occlusion patterns during training, but also receives explicit supervision on how much information remains visible in each local part, which provides a direct optimization signal for reliable part-aware aggregation.

### 3.5. Overall Training Objective

To jointly optimize global, occlusion-simulated, and part-level representations, we impose identity classification and metric learning losses on all feature branches. Given the fused global representation *z*, the two occlusion representations $z_{occ}^{\left(1\right)}$ and $z_{occ}^{\left(2\right)}$, and the part-level features ${\left\{z_{k}\right\}}_{k=1}^{K}$, we apply the cross-entropy loss [29] for identity supervision. For a feature embedding *u* with identity label *y*, the cross-entropy loss is defined as

(10) $$L_{\mathrm{id}}\left(u\right)=-\log\frac{e^{s_{y}}}{\sum_{c=1}^{C_{\mathrm{id}}}e^{s_{c}}},$$

where $C_{\mathrm{id}}$ denotes the number of training identities, and $s_{c}$ is the classification score of *u* for the *c*-th identity class. Accordingly, the overall identity classification loss is obtained by summing the cross-entropy losses over the fused global feature, the two occlusion-simulated features, and all part-level features:

(11) $$L_{\mathrm{id}}=L_{\mathrm{id}}\left(z\right)+L_{\mathrm{id}}\;\left(z_{\mathrm{occ}}^{\left(1\right)}\right)+L_{\mathrm{id}}\;\left(z_{\mathrm{occ}}^{\left(2\right)}\right)+\sum_{k=1}^{K}L_{\mathrm{id}}\left(z_{k}\right),$$

similarly, triplet loss [30] is applied to the corresponding feature embeddings to enforce metric consistency. For a feature embedding *u*, the triplet loss is defined as

(12) $$L_{\mathrm{tri}}\left(u\right)={\left[m+d\left(u,u^{+}\right)-d\left(u,u^{-}\right)\right]}_{+},$$

where $u^{+}$ and $u^{-}$ denote a positive sample with the same identity and a negative sample with a different identity, respectively, d(·, ·) denotes the Euclidean distance, *m* is the margin, and ${\left[\cdot \right]}_{+}=\max\left(0,\;\cdot \right)$. Accordingly, the overall triplet loss is computed over the fused global feature, the two occlusion-simulated features, and all part-level features:

(13) $$L_{\mathrm{tri}}=L_{\mathrm{tri}}\left(z\right)+L_{\mathrm{tri}}\;\left(z_{\mathrm{occ}}^{\left(1\right)}\right)+L_{\mathrm{tri}}\;\left(z_{\mathrm{occ}}^{\left(2\right)}\right)+\sum_{k=1}^{K}L_{\mathrm{tri}}\left(z_{k}\right),$$

the final training objective is formulated as

(14) $$L_{\mathrm{total}}=L_{\mathrm{id}}+L_{\mathrm{tri}}+\lambda_{\mathrm{vis}}L_{\mathrm{vis}},$$

where $\lambda_{vis}$ balances the contribution of the visibility supervision term. The optimal value of $\lambda_{vis}$ is empirically determined, and discussed in Section 4.6.4.

## 4. Experiments

### 4.1. Dataset Introduction

We evaluate the proposed VGOSL framework on five widely-used person re-identification benchmarks, including Occluded-Duke [11], DukeMTMC-reID [31], Market1501 [1], Partial-ReID [7], and MSMT17 [32].

**Occluded-Duke** is a challenging occlusion-focused benchmark derived from DukeMTMC-reID. It contains 15,618 training images of 702 identities. For evaluation, it includes 2210 occluded query images and 17,661 gallery images. It is specifically designed to assess person re-identification performance under severe occlusion.

**DukeMTMC-reID** is a large-scale person re-identification dataset collected from 8 cameras. It contains 36,411 images of 1404 identities in total. Following the standard DukeMTMC-reID setting, the training set includes 16,522 images of 702 identities. For evaluation, 17,661 images of another 702 identities are used for testing, including 2228 query images and the remaining gallery images. This benchmark is widely used for evaluating person re-identification performance under multi-camera scenarios.

**Market-1501** consists of 32,668 images of 1501 identities captured from 6 cameras. The training set contains 12,936 images of 751 identities, while the evaluation is conducted with 3368 query images and 19,732 gallery images from the remaining 750 identities.

**Partial-ReID** contains 600 images of 60 identities, with 5 holistic images and 5 partial images for each identity. During evaluation, partial images are used as probe images, while holistic images serve as gallery samples, making it suitable for assessing partial person matching performance.

**MSMT17** is a large-scale and challenging benchmark consisting of 126,441 images of 4101 identities captured from 15 cameras. The training set includes 32,621 images of 1041 identities, while the test set consists of 11,659 query images and 82,161 gallery images from 3060 identities.

### 4.2. Evaluation Metrics

Following standard ReID evaluation protocols, we adopt Cumulative Matching Characteristics (CMCs) [33] and mean Average Precision (mAP) [34] as the evaluation metrics. CMC measures whether at least one correct match appears within the top-k retrieved results, and Rank-1 accuracy is reported to reflect the probability that the correct identity is retrieved at the first position. In addition, mAP provides a more comprehensive evaluation of retrieval performance by considering both the precision and the ranking positions of all correct matches for each query. Therefore, CMC emphasizes top-rank matching capability, while mAP better reflects the overall ranking quality of the retrieval system.

### 4.3. Experimental Settings

All experiments are conducted on a server equipped with an NVIDIA vGPU with 32 GB of memory (Nvidia, Santa Clara, CA, USA). The models are implemented in PyTorch (2.3.0) and trained on a single GPU. We adopt a Transformer-based backbone instantiated by ViT-B/16 [6]. The stride size is set to 12 × 12. All input images are resized to 256 × 128 during both training and testing. Data augmentation includes random horizontal flipping (probability 0.5), padding of 10 pixels, and random erasing (probability 0.5). Pixel normalization is applied with mean [0.5, 0.5, 0.5] and standard deviation [0.5, 0.5, 0.5].

We employ the softmax-triplet sampler with 4 instances per identity in each mini-batch. The batch size is set to 32 for training and 256 for testing. The model is optimized using stochastic gradient descent with momentum 0.9 and weight decay $1\times 10^{-4}$. The initial learning rate is set to 0.008 and linearly warmed up at the beginning of training. The total training duration is 120 epochs, and evaluation is conducted every 10 epochs. The source code has been publicly released at: https://github.com/wangchunfengc/VGOSL (accessed on 10 May 2026).

### 4.4. Comparison with State-of-the-Art Methods

We compare the proposed VGOSL framework with recent state-of-the-art methods on six widely used benchmarks. For fair comparison, we report the results under the standard evaluation protocols of each dataset. The comparison is organized into three groups according to dataset characteristics.

#### 4.4.1. Duke-Family Benchmarks

DukeMTMC-reID and Occluded-Duke share the same identity pool and camera setup, where Occluded-Duke can be regarded as an occlusion-augmented variant of DukeMTMC-reID. Therefore, we group them together to evaluate both holistic and occluded scenarios under a consistent data distribution. As shown in Table 1, we compare VGOSL with recent transformer-based and occlusion-aware methods, including RTGAT [19], CTU [35], MSAMGO [36], CA-Jaccard [37], SSPEM [38], FCDA [39], IPCBReID [40], MTIPE [41], HDA-Net [42], FOSENet [43], POFR [44], OA-ReID [45] and MDFA [46]. On DukeMTMC-reID, VGOSL achieves competitive performance compared with these strong baselines. On the more challenging Occluded-Duke benchmark, VGOSL achieves competitive performance compared with recent state-of-the-art methods, especially in terms of mAP, demonstrating the effectiveness of part-aware visibility modeling under partial occlusion.

#### 4.4.2. Large-Scale Holistic Benchmarks

Market1501 and MSMT17 are large-scale holistic benchmarks that focus on identity scalability and diverse camera conditions rather than explicit occlusion modeling. We group them together to evaluate the generalization ability of VGOSL. Table 2 compares VGOSL with strong transformer-based baselines such as RTGAT [19], CTU [35], MSAMGO [36], CA-Jaccard [37], SSPEM [38], FCDA [39], MTIPE [41], HDA-Net [42], POFR [44], OA-ReID [45], COFD-Net [51], IPCBReID [40], MSPL [52], and MBDBFormer [53] VGOSL achieves competitive or superior results on both datasets, demonstrating that the proposed occlusion simulation and visibility-guided aggregation do not degrade holistic retrieval performance and maintain strong scalability on large identity sets.

#### 4.4.3. Small-Scale Partial Person Benchmarks

In addition to large-scale holistic datasets, we further evaluate VGOSL on small-scale partial person benchmarks, where probe images often contain severe occlusions or truncated body regions. Compared with conventional large-scale ReID datasets, these benchmarks are characterized by limited training samples and a higher degree of local information corruption, making them particularly challenging for robust feature learning. As reported in Table 3, we compare VGOSL with representative partial or occlusion-aware methods, including DSR [56], PGFA [11], PFT [57], CTU [35], POFR [44] and MSPL [52]. Despite the reduced data scale and increased difficulty, VGOSL consistently achieves competitive or superior performance across these benchmarks. This improvement can be attributed to the explicit modeling of part reliability and the adaptive reweighting mechanism, which suppress unreliable local responses and enhance discriminative visible regions. Such design enables VGOSL to construct more robust global representations under extreme partial observation conditions.

### 4.5. Ablation Study

#### 4.5.1. Module Ablation

To verify the contribution of each core module to the overall performance, we conduct module ablation experiments on the Occluded-Duke dataset, and the results are reported in the Table 4. The baseline model achieves 54.1% mAP and 65.0% Rank-1. After introducing the PVM module, the performance improves significantly to 61.8% mAP and 71.5% Rank-1, corresponding to gains of 7.7 percentage points and 6.5 percentage points over the baseline, respectively. Furthermore, when the OBS module is individually added to the baseline, the model achieves 62.0% mAP and 71.8% Rank-1, which are 7.9 percentage points and 6.8 percentage points higher than the baseline. When both PVM and OBS are jointly introduced, the model further improves to 63.1% mAP and 72.8% Rank-1, achieving the best performance among all configurations. Compared with the baseline, the overall improvement reaches 9.0 percentage points in mAP and 7.8 percentage points in Rank-1. These results indicate that PVM and OBS exhibit good complementarity. Overall, both modules independently bring stable performance gains, while their combination yields the best results, thereby validating the effectiveness and rationality of the proposed design.

#### 4.5.2. Ablation on the Number of Partitions

To analyze the effect of the partition number on non-model performance, we conduct ablation experiments with different partition settings on the Occluded-Duke dataset, and the results are reported in Table 5. When *N* = 1, the model achieves 62.6% mAP and 70.6% Rank-1, indicating that coarse-grained local modeling alone is still insufficient for capturing fine-grained discriminative cues under occlusion. As the number of partitions increases, the overall performance shows an initial improvement followed by slight fluctuations. When *N* = 2, the model reaches 62.7% mAP and 71.5% Rank-1. Further increasing the number to *N* = 3 improves the mAP to 63.2%, while the Rank-1 slightly drops to 71.4%. When *N* = 4, the model achieves 63.1% mAP and 72.8% Rank-1, yielding the best Rank-1 performance among all settings while maintaining a high mAP level. Although the mAP at *N* = 3 is 0.1 percentage points higher than that at *N* = 4, its Rank-1 is noticeably lower. This suggests that a smaller number of partitions may offer a marginal advantage in overall retrieval precision, but it is less effective in terms of the most critical top-1 matching accuracy. When the partition number is further increased to *N* = 5 and *N* = 6, the performance no longer improves and instead declines to 62.8%/72.1% and 63.0%/71.4%, respectively. This indicates that excessive partitioning may overly fragment local regions, weakening the semantic completeness contained in each part and introducing more noise and instability, which is unfavorable for robust feature learning.

Overall, an appropriate partition strategy helps enhance the modeling of locally visible regions and thus improves discriminative robustness in occluded scenarios. As shown in Table 5, increasing the number of partitions generally brings performance gains by enabling finer-grained local representation learning. When comparing N=3 and N=4, the mAP of N=4 is only 0.1 percentage points lower than that of N=3, while its Rank-1 accuracy is improved by 1.4 percentage points. This indicates that N=4 achieves a better balance between overall retrieval accuracy and top-ranked matching performance. Therefore, we finally choose N=4 as the optimal partition setting.

### 4.6. Visualization Analysis

#### 4.6.1. t-SNE Feature Distribution Visualization Analysis

To provide an intuitive understanding of the discriminative ability of the learned features, we perform t-SNE visualization on the extracted embeddings. Specifically, we randomly select 30 person identities from the test set and project their high-dimensional features into a two-dimensional space for comparison between the baseline and the proposed method.

**Figure 4:**
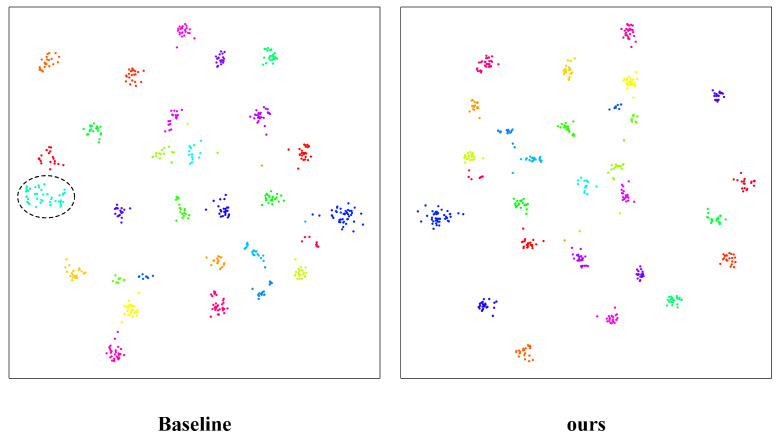
t-SNE visualization of feature embeddings for the baseline and the proposed method. Different colors denote different person identities in the embedding space. The circles indicate regions where inter-class distinctions are ambiguous.

As shown in Figure 4, the feature distributions produced by the baseline exhibit noticeable overlap among several identities. In particular, the samples highlighted by the dashed circle are mixed together in the embedding space, indicating that the baseline fails to sufficiently separate some visually similar or heavily occluded identities. Such ambiguous decision boundaries suggest limited discriminative power under challenging occlusion scenarios.

In contrast, the feature embeddings generated by our method form more compact intra-class clusters and clearer inter-class separations. Most identities are well grouped with reduced overlap between different categories, demonstrating that the proposed method learns more discriminative and robust representations. This improvement can be attributed to the enhanced modeling of visible local regions and the strengthened ability to suppress interference caused by occluded or noisy regions.

Overall, the t-SNE visualization qualitatively verifies that our method produces a more separable feature space than the baseline, which is consistent with the quantitative improvements observed in the retrieval results.

#### 4.6.2. Retrieval Visualization Analysis

**Figure 5:**
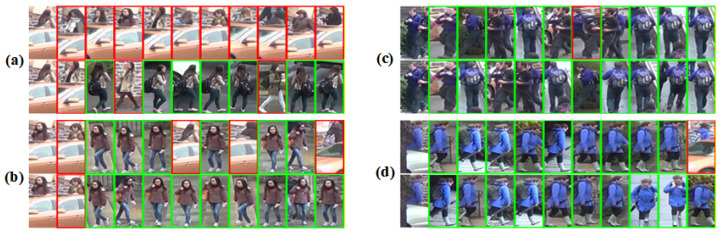
Visualization of top-ranked retrieval results for the baseline and the proposed method. In each group, the first row corresponds to the baseline, while the second row corresponds to the proposed method. Green borders denote correct matches, whereas red borders indicate incorrect retrievals. Panels (**a**–**d**) present four representative retrieval cases.

To further evaluate the practical retrieval behavior of the proposed method, we visualize the top-ranked retrieval results of the baseline and our method on the test set, as shown in Figure 5. Specifically, we present two successful cases in (c) and (d), as well as two failure cases in (a) and (b). In each group, the first row corresponds to the baseline, while the second row corresponds to our method. The images with green borders denote correct matches, whereas those with red borders indicate incorrect retrievals.

As shown in the successful cases, our method generally yields more accurate and stable retrieval results than the baseline. In particular, in case (d), the proposed method demonstrates a stronger ability to capture discriminative fine-grained details under challenging conditions. Although the probe image suffers from occlusion, our method still retrieves more correct matches at higher ranks, while the baseline makes an incorrect Rank-1 prediction under severe occlusion. This suggests that the proposed method is more effective in focusing on informative visible regions and suppressing interference from occluded or irrelevant areas.

At the same time, the failure cases further reveal the limitations of both methods in extremely challenging scenarios. In case (a), although our method achieves a noticeably higher retrieval success rate than the baseline overall, incorrect matches still appear in the top-ranked results. A closer inspection shows that, under severe occlusion, the wrongly retrieved samples share highly similar local appearance details with the query, which makes them difficult to distinguish. This indicates that when only limited visual cues are available, both methods may still confuse identities with highly similar fine-grained characteristics.

Overall, the retrieval visualizations demonstrate that the proposed method improves the ranking quality and matching robustness compared with the baseline, especially in occluded scenarios. Nevertheless, the failure examples also suggest that there is still room for improvement in finer-grained identity discrimination, particularly when different pedestrians exhibit highly similar local textures, poses, or visible body parts under heavy occlusion.

#### 4.6.3. Intra-Class/Inter-Class Distance Distribution Analysis

**Figure 6:**
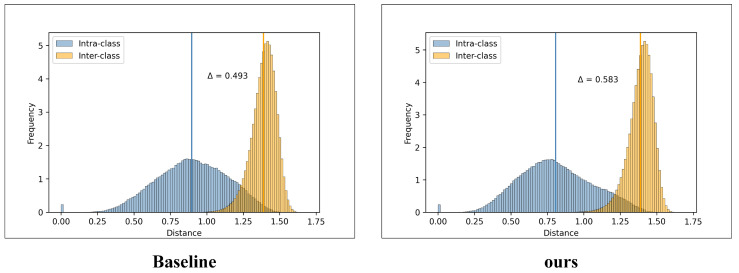
Visualization of intra-class and inter-class distance distributions for the baseline and the proposed method. The blue and orange histograms denote the intra-class and inter-class distance distributions, respectively, and Δ indicates the separation margin between the two distributions.

To further investigate the discriminative capability of the learned features, we visualize the distributions of intra-class and inter-class distances for the baseline and the proposed method. As shown in Figure 6, the blue histogram denotes the intra-class distance distribution, while the orange histogram represents the inter-class distance distribution.

From the baseline distribution, although the intra-class and inter-class distances are generally separable, there still exists a noticeable overlap region between the two distributions. This indicates that the baseline may still confuse some hard positive and hard negative samples, leading to limited discriminative power in challenging scenarios. In contrast, the proposed method exhibits a clearer separation between the two distributions. The intra-class distances are more concentrated in a lower-distance region, while the inter-class distances are distributed farther away, resulting in a larger margin between the two groups.

In particular, the distance gap Δ increases from 0.493 in the baseline to 0.583 in our method. The enlarged separation margin suggests that the proposed model is able to pull samples from the same identity closer together while pushing samples from different identities farther apart. Therefore, the learned embedding space becomes more discriminative and structured, which directly reflects the stronger class separability of our method.

Overall, the intra-class/inter-class distance visualization provides intuitive evidence that the proposed method achieves higher inter-class discrimination and better intra-class compactness than the baseline, thereby contributing to more reliable person re-identification under challenging conditions.

#### 4.6.4. Hyper-Parameter Sensitivity Analysis

**Figure 7:**
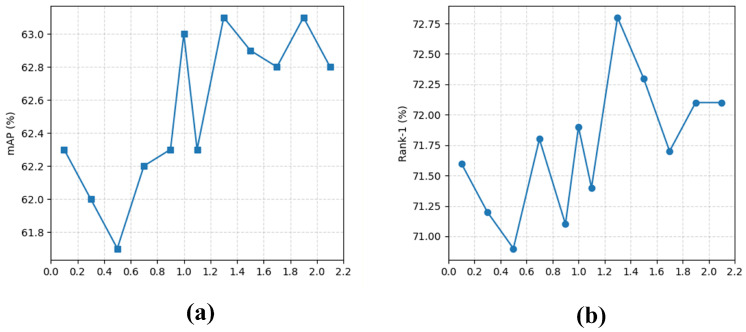
Hyper-parameter analysis of $\lambda_{vis}$. (**a**) mAP under different $\lambda_{vis}$ values. (**b**) Rank-1 under different $\lambda_{vis}$ values.

To analyze the effect of the hyper-parameter $\lambda_{vis}$, we vary it from 0.1 to 2.1 and report the corresponding mAP and Rank-1 results in Figure 7. It can be observed that the model achieves the best performance when $\lambda_{vis}$ = 1.3. Meanwhile, the performance remains relatively stable across the entire interval, without obvious fluctuations. This indicates that the proposed method is robust to the choice of $\lambda_{v}is$ and does not require highly sensitive hyper-parameter tuning. Therefore, $\lambda_{vis}$ = 1.3 is selected as the default setting in our experiments.

#### 4.6.5. Model Complexity Analysis

To further analyze the complexity of the proposed method, we compare different models in terms of parameter size and FLOPs, as shown in the Table 6. It can be observed that our method contains 93.090 M parameters, which is still relatively compact compared with some existing methods. This indicates that, although additional occlusion-aware and visibility-related modules are introduced, the overall parameter scale remains at an acceptable level without causing excessive parameter growth.

Meanwhile, the FLOPs of our method increase to 64.1314 G, compared with 20.8005 G for the baseline. The main reason for this increase is not that the backbone itself becomes excessively complicated, but that our method adopts a multi-input strategy during the forward process. Specifically, the model takes the original image, cropped image, and randomly erased image as inputs simultaneously. Since all three inputs need to pass through feature extraction and subsequent representation learning stages, the overall computational cost increases substantially. Therefore, the rise in FLOPs is mainly caused by the use of multiple input branches rather than by a significant expansion of the model parameters.

Despite the increased computational cost, our method achieves better retrieval performance, reaching 72.8% Rank-1 and 63.1% mAP. This suggests that the additional computation is effectively used to enhance occlusion modeling and fine-grained feature representation, thereby leading to more discriminative and robust retrieval results. Overall, the proposed method achieves a favorable balance among model size, computational complexity, and recognition performance.

To further verify whether the multi-input strategy is necessary during testing, we additionally evaluate an original-only inference setting. Specifically, the final trained weights are loaded, while only the original image is used during inference. As shown in Table 6, the original-only setting requires only one forward pass per image and reduces the inference time from 6.783 ms/img to 3.541 ms/img. Meanwhile, it achieves the same Rank-1 accuracy of 72.8% and mAP of 63.1% as the multi-input setting. This indicates that the occlusion-simulated inputs mainly serve as an auxiliary regularization strategy during training, helping the original-image branch learn more robust occlusion-aware representations. Therefore, the proposed method can be simplified to original-only testing for practical deployment without requiring multiple forward passes at test time.

#### 4.6.6. Heatmap Visualization Analysis

**Figure 8:**
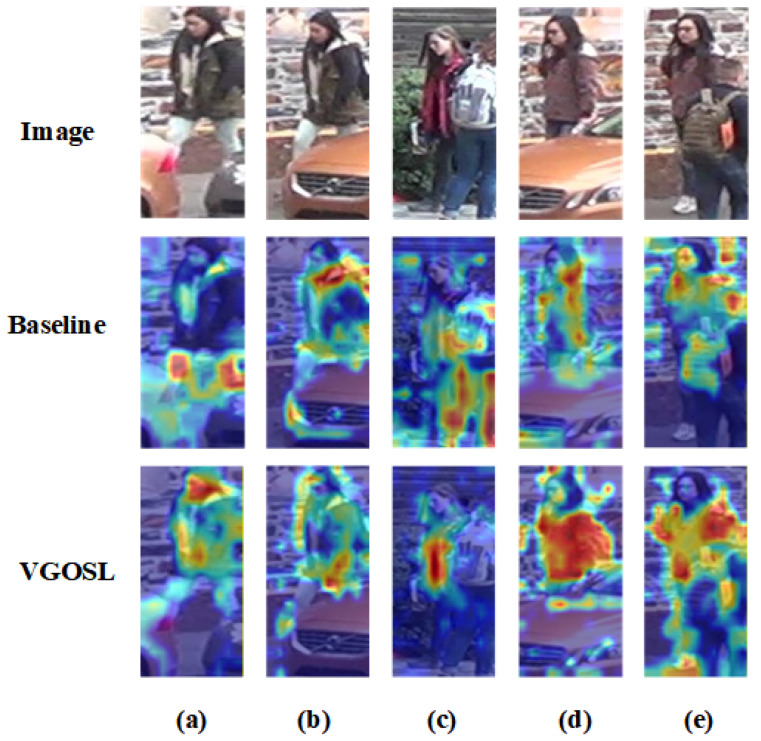
Visualization of activation heatmaps for the baseline and the proposed VGOSL on representative occluded person images. The first row shows the input images, while the second and third rows present the heatmaps generated by the baseline and VGOSL, respectively. It can be observed that VGOSL focuses more accurately on the target pedestrian regions and is less affected by surrounding occlusions or irrelevant background interference. Panels (**a**–**e**) present five representative visualization cases.

To further investigate the attention behavior of the model under occluded conditions, we visualize the activation heatmaps of the baseline and VGOSL on several representative examples, as shown in Figure 8. From the figure, it can be observed that our method exhibits more accurate and stable responses to discriminative pedestrian regions.

For cases (a), (b), and (d), the upper body of the target pedestrian remains largely visible without severe occlusion. In these examples, the heatmaps generated by VGOSL focus more consistently on the overall human body regions, especially the visible upper-body structure and appearance cues. Compared with the baseline, our method produces more concentrated and semantically meaningful activations, indicating a stronger ability to capture informative pedestrian features rather than irrelevant background responses.

For cases (c) and (e), the target pedestrian is partially occluded by other nearby persons, making the identification task more challenging. Under such conditions, the baseline is more easily distracted by surrounding pedestrians, and part of its attention is shifted to interference regions, which may weaken the discriminative representation of the target identity. In contrast, VGOSL can still accurately focus on the target pedestrian and emphasize the visible local body parts that remain informative. This suggests that our method is better at suppressing interference from occluding persons and exploiting local visible cues for robust person identification.

Overall, the heatmap visualizations demonstrate that the proposed method can attend to more relevant pedestrian regions under both mild and severe occlusion. Especially in crowded or partially occluded scenarios, it shows stronger target awareness and local discriminative modeling ability, which further explains its superior retrieval performance.

## 5. Conclusions

In this paper, we propose an occlusion-aware person re-identification framework that enhances the modeling of visible local regions and improves the robustness of feature representation under occluded conditions. Extensive experiments demonstrate that the proposed method effectively improves retrieval performance and feature discriminability compared with the baseline. In particular, the proposed PVM module explicitly models part-level visibility reliability and guides adaptive global feature aggregation, while the OBS module improves robustness through structured occlusion simulation during training. In addition, the ablation studies and visualization results further verify the effectiveness of the proposed design. Although our method can accurately focus on informative local cues, it may still face challenges when different pedestrians exhibit highly similar local appearances under severe occlusion. Therefore, our future work will focus on more fine-grained local feature learning and finer-detail discrimination to further improve recognition performance in highly ambiguous occluded scenarios.

## Figures and Tables

**Table 1:** Comparison with state-of-the-art methods on Occluded-Duke and DukeMTMC-reID. “-” indicates that the corresponding result was not reported in the original paper. Bold numbers indicate the best performance.

Method	Venue	Occluded-Duke	DukeMTMC-reID
		R-1/mAP	R-1/mAP
OPR-DAAO [47]	TIFS’22	66.2/55.4	88.5/76.5
RTGAT [19]	TIP’23	61.0/50.1	89.1/80.2
BPBreID [48]	WACV’23	68.3/55.2	89.6/78.3
PRE-Net [49]	TCSVT’23	66.7/54.1	88.9/76.5
MHSA-Net [50]	TNNLS’23	55.4/42.4	87.3/73.1
CTU [35]	TBIOM’24	68.2/56.1	89.5/78.3
MSAMGO [36]	ESWA’24	-	86.8/76.9
CA-Jaccard [37]	CVPR’24	-	-
SSPEM [38]	ESWA’24	70.2/62.8	-
FCDA [39]	ESA’25	60.9/50.6	88.1/76.7
IPCBReID [40]	EAAI’25	66.1/54.8	-
MTIPE [41]	PR’25	66.4/57.8	90.1/80.8
HDA-Net [42]	ICETCI’25	69.2/61.3	89.7/78.8
FOSENet [43]	PR’25	68.7/61.5	90.8/83.8
POFR [44]	IJON’25	72.9/62.0	90.4/82.5
OA-Rel [45]	Inf. Sci.’25	**74.2**/62.4	90.4/81.2
MDFA [46]	DSP’26	69.6/62.4	91.2/**83.9**
Ours	-	72.8/**63.1**	**91.2**/81.3

**Table 2:** Comparison with state-of-the-art methods on Market-1501 and MSMT17. “-” indicates that the corresponding result was not reported in the original paper.

Method	Venue	Market-1501	MSMT17
		R-1/mAP	R-1/mAP
DRL-Net [54]	TMM’22	94.7/86.9	78.4/55.3
RTGAT [19]	TIP’23	95.3/88.2	-
RTGAT [19]	TIP’23	95.3/88.2	-
TR-AMG [55]	TMM’23	68.5/59.7	-
SSPEM [38]	ESA’24	95.3/89.2	84.0/64.6
CTU [35]	TBIOM’24	95.7/88.3	-
MSAMGO [36]	ESWA’24	94.7/87.2	68.5/42.5
CA-Jaccard [37]	CVPR’24	94.4/86.1	75.1/44.3
SSPEM [38]	ESWA’24	95.3/89.2	84.0/64.6
FCDA [39]	ESA’25	94.7/85.4	-
MTIPE [41]	PR’25	95.2/89.0	80.4/61.1
HDA-Net [42]	ICETCI’25	95.4/87.9	-
POFR [44]	IJON’25	95.1/87.5	-
OA-RelD [45]	Inf. Sci.’25	95.8/89.1	-
COFD-Net [51]	DSP’25	95.9/89.5	85.9/67.6
IPCBReID [40]	EAAI’25	**96.8**/89.5	-
MSPL [52]	IJON’25	95.6/89.8	84.8/65.7
MBDBFormer [53]	DSP’26	95.6/90.0	**87.2/70.8**
Ours	-	95.8**/90.0**	87.0/70.5

**Table 3:** Comparison with state-of-the-art methods on Partial-ReID. “-” indicates that the corresponding result was not reported in the original paper.

Method	Venue	Partial-ReID
		R-1/R-3
DSR [56]	ESA’18	58.8/67.2
PGFA [11]	CVPR’19	69.1/80.9
PVPM [58]	CVPR’20	78.3/87.7
PFT [59]	TSCVT’20	81.3/-
PFT [57]	NCA’22	81.3/-
CTU [35]	TBIOM’24	85.7/91.7
POFR [44]	IJON’25	**89.3**/91.3
MSPL [52]	IJON’25	87.6/92.2
Ours	-	84.0**/92.3**

**Table 4:** Ablation study of different modules on Occluded-Duke. “✓” indicates that the corresponding module is used, while “-” indicates that the module is not used.

Baseline	PVM	OBS	mAP/Rank-1
✓	-	-	54.1/65.0
✓	✓	-	61.8/71.5
✓	-	✓	62.0/71.8
✓	✓	✓	**63.1**/**72.8**

**Table 5:** Ablation study on the number of partitions *N* on Occluded-Duke.

*N*	mAP/Rank-1
1	62.6/70.6
2	62.7/71.5
3	**63.2**/71.4
4	63.1/**72.8**
5	62.8/72.1
6	63.0/71.4

**Table 6:** Model complexity comparison. “-” indicates that the corresponding result was not reported in the original paper. Time denotes the average inference time per image.

Method	Params (M)	FLOPs (G)	Time (ms/img)	Rank-1 (%)	mAP (%)
HDA-Net [42]	101.800	-	-	69.2	61.2
baseline	85.842	20.8005	2.205	65.0	54.1
Baseline + OBS	92.940	64.1308	6.790	71.8	62.0
ours	93.090	64.1314	6.783	72.8	63.1
Original-only	93.090	22.53	3.541	72.8	63.1

## Data Availability

The datasets used in this study (Occluded-DukeMTMC, DukeMTMC-reID, Market-1501, Partial-ReID, and MSMT17) are publicly available. No new data were created or analyzed in this study.

## Abbreviations

The following abbreviations are used in this manuscript:

ReID	Person Re-Identification
PVM	Part-aware Visibility Modeling
OBS	Occlusion Box Simulation
VGOSL	Visibility-Guided and Occlusion-Simulated Learning
ViT	Vision Transformer
CNN	Convolutional Neural Network
MLP	Multi-Layer Perceptron
BCE	Binary Cross-Entropy
mAP	Mean Average Precision
Rank-1	Rank-1 Accuracy
Occluded-DukeMTMC	Occluded-DukeMTMC-reID Dataset
DukeMTMC-reID	Duke Multi-Target Multi-Camera Person Re-Identification Dataset
Market-1501	Market-1501 Person Re-Identification Dataset
Partial-ReID	Partial Person Re-Identification Dataset
MSMT17	Multi-Scene Multi-Time Person Re-Identification Dataset
